# Stress-Related Methylation Risk Scores Predict Coronary Heart Disease

**DOI:** 10.64898/2026.07.31.26359423

**Published:** 2026-08-03

**Authors:** Sofia Benavides, Hazel Milla, Helena Palma-Gudiel, David Checknita, Bjoernar Tuftin, Kai Xia, Charles Kooperberg, Alexander P. Reiner, JoAnn E. Manson, Themistocles L. Assimes, Parveen Bhatti, Kent D. Taylor, W. Craig Johnson, Stephen S. Rich, Jerome I. Rotter, Linda C. Gallo, David R. Rubinow, Elior Rahmani, Laura M. Raffield, Eric A. Whitsel, Anthony S. Zannas

## Abstract

**Background:**

Psychosocial stress is a key risk factor for coronary heart disease (CHD), particularly in postmenopausal women who face both a high stress burden and elevated cardiovascular risk. DNA methylation (DNAm) – a critical epigenetic modification bridging environment and health – remains understudied as a contributor to stress- related CHD.

**Methods:**

We conducted an epigenome-wide association study (EWAS) of stress in the Women’s Health Initiative (WHI), an ancestrally diverse cohort of postmenopausal women (n=3,857). At screening visit, participants completed a questionnaire assessing stressful life events and provided whole blood for DNAm. Incident CHD was then longitudinally ascertained (follow-up mean/SD: 16.7/8.4 years), and DNAm signatures were evaluated as CHD predictors using Cox regression. Predictive models were independently validated in the Jackson Heart Study (JHS; n=3,053) and Multi-Ethnic Study of Atherosclerosis (MESA; n=870). The bulk-level DNAm associations were computationally deconvolved at the cell-type-specific level using tensor composition analysis (TCA).

**Results:**

The EWAS in WHI identified 841 stress-related DNAm sites (99 hypermethylated, 742 hypomethylated with stress) after FDR correction, with 13 significant after Bonferroni correction, including sites located on immune and CHD- related genes (e.g., *TNF* , *ALDH2* ). Methylation risk scores (MRSs) integrating the 841 FDR-significant sites (MRS _841_ ) and 13 Bonferroni-significant sites (MRS _13_ ) predicted incident CHD (HR=1.33-1.37; p≤0.0008) and mediated 16.5-17.7% of the association between stress and CHD. In JHS and MESA, MRS _13_ independently predicted CHD (HR=1.34; p=0.036), whereas MRS _841_ was suggestively associated with CHD (HR=1.27; p=0.087). TCA indicated that the greatest number of stress-related sites predictive of CHD was specifically in monocytes (133 total), with directions consistent with bulk-level associations (9 hypermethylated, 124 hypomethylated with stress).

**Conclusion:**

Our study supports methylation risk scores as novel biomarkers of stress- related CHD and uncovers epigenetic regulation in monocytes as a potential underlying mechanism. These findings highlight biological pathways linking stress and disease and may promote personalized interventions in high-risk populations.

## Full Text Availability

The license terms selected by the author(s) for this preprint version do not permit archiving in PMC. The full text is available from the preprint server.

